# Within-host adaptation mutations associated with persistent colonization of *Pseudomonas aeruginosa* in a silicosis patient

**DOI:** 10.3389/fcimb.2026.1739179

**Published:** 2026-02-17

**Authors:** Furong Zhang, Lvxin Qian, Yi Yan, Qiuling Wan, Luhua Zhang, Ying Li

**Affiliations:** 1 Department of Clinical Laboratory, Pengzhou People’s Hospital, Chengdu, Sichuan, China; 2 The School of Basic Medical Sciences, Southwest Medical University, Luzhou, Sichuan, China

**Keywords:** adaptive strategies, biofilm formation, imipenem-resistant, mucoid, *P. aeruginosa*

## Abstract

*Pseudomonas aeruginosa* is a notorious opportunistic pathogen that causes life-threatening infections in immunocompromised individuals. In this study, we isolated two mucoid imipenem-resistant *P. aeruginosa* strains, SCPa14 and SCPa16, from a single silicosis patient. Whole-genome sequencing revealed that these two strains are homologous, both of which belong to the epidemic high-risk clone ST274. Gene sequence analysis identified a deletion mutation (ΔG 433) of the *mucA* gene and a premature stop mutation in the *oprD* gene in SCPa14 and SCPa16; these mutations are assumed to be responsible for their mucoid phenotype and imipenem resistance, respectively. Compared to the reference strain PAO1, both strains showed slow growth, reduced cell motility, increased pyocyanin production, and enhanced H_2_O_2_ resistance, which may collectively aid their adaptation to the respiratory tract environment. SCPa14 had impaired biofilm formation, which may be attributed to a premature termination mutation in its *relA* gene. In contrast, SCPa16 exhibited enhanced biofilm formation—a trait that may explain its persistence within the host. However, both strains showed poor growth under iron-restriction stress, which likely results from their lack of pyoverdine genes; they also exhibited impaired resistance to the antimicrobial peptide LL-37 and serum complement. Consistent with these traits, SCPa14 and SCPa16 were identified as low-virulence strains using the *G. mellonella* infection experiment. Overall, these data improve understanding of the adaptive strategies and fitness costs of *P. aeruginosa* during chronic infection in the silicosis patient, which offers new insights for antimicrobial therapy.

## 1 Introduction

*Pseudomonas aeruginosa* is a ubiquitous environmental Gram-negative bacterium, and also one of the most feared opportunistic pathogens capable of causing a range of acute and chronic infections (Folkesson et al., 2012). It is a common pathogen causing chronic respiratory infections in patients with underlying conditions, such as cystic fibrosis (CF) and chronic obstructive pulmonary disease (Lau et al., 2004). *P. aeruginosa* is also a notorious pathogen notable for its remarkable adaptability and its ability to evade host immune defenses. It possesses a large genome and high genetic plasticity that enable it to rapidly adapt to diverse ecological niches, and the formation of biofilms promotes persistence and evasion of phagocytic clearance (Kung et al., 2010; Jangra et al., 2022). Treating *P. aeruginosa* infections is extremely difficult due to its rapid adaptive mutations and acquired antibiotic resistance (Jangra et al., 2022; Qin et al., 2022). Carbapenem-resistant *P. aeruginosa* is listed among the “high” group of priority pathogens by WHO (List, 2024), highlighting that novel alternative strategies are urgently needed.

During the course of chronic respiratory infections, *P. aeruginosa* colonizers often convert from non-mucoid to a mucoid phenotype owing to excessive and constitutive production of the extracellular polysaccharide alginate (Qiu et al., 2007). Alginate overproduction is often interpreted as a sign of biofilm development, which plays a key role in protecting bacteria from both host defenses and antibiotic treatment (Thi et al., 2020). Clinical isolation of mucoid *P. aeruginosa* often indicates increased infection complexity, elevated treatment difficulty, and poor prognosis in specific patients (Qiu et al., 2007). These mucoid *P. aeruginosa* isolates are often accompanied by other phenotypic changes, such as slow growth, antibiotic resistance, loss of motility, and inhibition of quorum sensing (Rossi et al., 2021). These phenotypic changes and their linked genetic mutations are a result of bacteria’s parallel evolution within the host, and they are helpful for bacterial *in vivo* growth and survival.

Silicosis is a chronic fibrotic lung disease resulting from the inhalation of free crystalline silicon dioxide, also known as silica (Leung et al., 2012). Pulmonary structural damage and impaired immune function caused by silicosis significantly increase the risk of bacterial infection, which in turn exacerbates the condition of silicosis, forming a “damage-infection-exacerbation” vicious cycle (Leung et al., 2012). The adaptation of *P. aeruginosa* to the CF lung has been extensively studied, while studies on its evolutionary adaptation in silicosis patients remain rare. In this study, two mucoid imipenem-resistant *P. aeruginosa* were isolated from the same silicosis patient. We conducted whole-genome sequencing and phenotypic analysis to characterize their genetic and phenotypic changes. The findings highlight adaptive evolution of *P. aeruginosa* strains within the host and their accompanying fitness costs.

## 2 Materials and methods

### 2.1 Patient data, strain isolation, and growth conditions

A 70-year-old male silicosis patient was admitted to Pengzhou People’s Hospital in Sichuan Province, China, on April 16th, 2024, due to recurrent cough, expectoration of sputum, and fatigue. He was diagnosed with chronic obstructive pulmonary disease (COPD) and bacterial pneumonia. During hospitalization, the patient was administered ciprofloxacin to initiate anti-infection treatment. The sputum samples were inoculated onto the blood agar plate, chocolate agar plate, and MacConkey agar plate (all supplied by Antu Biotechnology, China), respectively. Then, blood agar plates and chocolate agar plates were incubated in a 35°C, 5% CO_2_ incubator, while the MacConkey agar plate was cultured in a 35°C conventional incubator. Bacterial growth was monitored at 24 and 48 h post-incubation. Once suspicious colonies appeared, the isolates were purified by subculturing on blood agar plates. Following morphological assessment of colonies on the plates, a single colony was randomly selected if uniform morphology without significant variation was observed. Two bacterial isolates (designated SCPa14 and SCPa16) were obtained from the sputum sample at two different sampling times: SCPa14 was recovered on the day of admission, while SCPa16 was isolated 9 days later. The strains were identified as *P. aeruginosa* with the AutoFMS600 system using MALDI-TOF MS technology (Autobio Diagnostics CO., Ltd, China). All bacterial strains were cultured using LB medium at 37°C with shaking (200 rpm), unless stated otherwise.

### 2.2 Antimicrobial susceptibility testing

*In vitro* antimicrobial susceptibility tests were conducted by disk diffusion method on Müller-Hinton (MH) agar plates according to recommendations of the Clinical and Laboratory Standards Institute (CLSI, M100-33th). The antibiotics tested were the following: piperacillin, piperacillin/tazobactam, cefoperazone/sulbactam, gentamicin, ceftazidime, cefepime, aztreonam, imipenem, meropenem, tobramycin, amikacin, levofloxacin, and ciprofloxacin. Results were interpreted following the CLSI guidelines. *P. aeruginosa* ATCC 27853 and *Escherichia coli* ATCC 25922 strains served as quality control for antimicrobial susceptibility testing.

### 2.3 Whole-genome sequencing and bioinformatics analysis

WGS of SCPa14 and SCPa16 was conducted using the HiSeq 2000 Sequencer with a 150 bp paired-end library and 200 × coverage by the Tsingke Biotechnology Co., Ltd. Raw reads were trimmed with Trimmomatic v0.38 (Bolger et al., 2014), followed by assembly into draft genomes using SPAdes v3.12.0 (Bankevich et al., 2012). SCPa14 was also subjected to WGS using Nanopore PromethION combined with Illumina NovaSeq PE150 platform by the Novogene Co., Ltd. Assembly of the second- and third-generation sequencing data was performed using Unicycler software v0.4.3 (Wick et al., 2017). Genome annotation was carried out using Prokka software v1.14 (Seemann, 2014). Average nucleotide identity (ANI) analysis was conducted using the ANI Calculator (Yoon et al., 2017). Antibiotic resistance genes (ARGs) and virulence genes were predicted using ResFinder database (Bortolaia et al., 2020) and Virulence Factor Database (VFDB) (Chen et al., 2005), respectively. Single-nucleotide polymorphism (SNP) analysis was performed by aligning the genome sequence of SCPa16 with that of SCPa14 using Breseq version 0.39.0 (Deatherage and Barrick, 2014). Gene sequence alignment was performed using the SnapGene tool (GSL Biotech, http://www.snapgene.com/).

### 2.4 Growth assay

An overnight culture was inoculated into fresh LB broth at an initial optical density at 600 nm (OD600) of ~0.1, in a final volume of 200 μL. The culture was then grown at 37°C for 24 h in 96-well plates, with a shaking cycle every 60 min when OD600 was measured by a microplate reader (BioTek, Synergy H1). This experiment was independently performed three times in triplicate.

### 2.5 Biofilm quantification

Biofilm formation ability was determined by crystal violet staining as previously described with some modifications (Fu et al., 2025). Briefly, log-phase culture of *P*. *aeruginosa* strains was adjusted to an OD600 of ~0.1 and diluted at 1:100 into fresh LB broth, followed by inoculating statically in sterile 96-well microtiter plates (200 μL/well) at 37°C. After 48 h, 100 μL of the bacterial suspension was collected from each well, serially diluted, and plated onto LB agar plates; the number of bacterial colony-forming units (CFUs) was enumerated after overnight incubation at 37°C. Meanwhile, the remaining culture in each well was aspirated, and the wells were washed three times with sterile water. After drying, the biofilm in each well was fixed by adding 200 μL of methanol. After 15 min, the methanol was removed, and each well was stained with 0.1% (w/v) crystal violet. Following 15 min of staining, the crystal violet solution was aspirated, and the wells were rinsed with sterile water. The bound dye was solubilized by adding 200 μL of 36% (v/v) acetic acid. The biofilm formation level was determined by measuring the absorbance at 595 nm using a spectrophotometer.

### 2.6 Motility assays

Swimming and swarming were determined as described previously (Déziel et al., 2001). For the swimming assay, 2 μL aliquots of the overnight culture of *P*. *aeruginosa* strains were dropped onto the surface of tryptone swim plates (10 g/L tryptone, 5 g/L NaCl, 0.3% agar), followed by incubation for 18 h at 37°C. For the swarming assay, 2 μL aliquots of overnight culture of *P*. *aeruginosa* strains were dropped onto the swarm plates that composed of nutrient broth (8 g/L), glucose (2.5 g/L), and agar (0.3%). The plates were incubated at 37°C for 24 h. Motility was then assessed qualitatively by observing the circular turbid zone formed as bacterial cells migrated away from the inoculation point.

### 2.7 Pyocyanin and pyoverdine production

Pyocyanin production was measured by extracting pyocyanin from the bacterial supernatant using chloroform and HCL, as previously described with minor modifications (Zhu et al., 2024). Cultures of *P*. *aeruginosa* strains were grown to the late log phase, and their OD_600_ values were measured using a spectrophotometer. The bacterial cultures were sedimented by centrifugation, and 1.8 mL of chloroform was added to 3 mL of the supernatant, followed by thorough mixing. After that, 1.5mL of the chloroform layer was transferred into a new centrifuge tube and mixed with 0.5 mL of 0.2 M HCL. After vigorous vortexing, the mixture was centrifuged again at 12,000 rpm for 2 minutes to collect the acidic aqueous supernatant. The absorbance of this supernatant at 520 nm (A_520_) was determined. The pyocyanin concentration= (*A*520×17.072)/initial OD_600_.

Pyoverdine production was assayed as previously described (La Rosa et al., 2021). Cells were cultivated in 3 mL of King’s B medium at 37 °C for 48 h. The relative concentration of pyoverdine in the supernatants was quantified by measurement of the fluorescence at 460 nm after excitation at 400 nm with a Synergy H1 Hybrid Multi-Mode Reader (BioTek, USA). The pyoverdine concentration was quantified as relative fluorescence units (RFU) normalized to the absorbance of the cell culture (OD_600_). The experiments were performed three times independently with at least three technical replicates per experiment.

### 2.8 Iron-limited assay

Overnight culture of *P*. *aeruginosa* strains was serially diluted with sterile PBS, 5 μL aliquots of the diluted solution (10^2^, 10^3^, 10^4^, 10^5^, 10^6^, or 10^7^ CFU/mL) were dropped onto the surface of iron-limited LB agar plates (containing 300 μM 2,2’-dipyridyl). The plates were incubated at 37°C for 16–18 h.

### 2.9 qRT-PCR

qRT-PCR was performed as we described previously (Qiu et al., 2024). *P*. *aeruginosa* strains were cultured in drug-free LB broth and grown to the mid-log phase (OD_600_~0.6-0.8). Total RNA was isolated from bacteria using the Bacterial RNA Isolation Kit (GBCBIO, China). The cDNAs used for PCR were synthesized with the PrimeScript™ RT reagent Kit with gDNA Eraser (TaKaRa, Japan), according to the manufacturer’s instructions. The primers for qRT-PCR are listed in **Supplementary Table S1**. qRT-PCR amplification was conducted using the LineGene 9600 Plus PCR Detection System (BIOER, China). The RNA samples were normalized to the level of the housekeeping gene *rpsL*. Relative gene expressions were calculated using the 2^-ΔΔCT^ method. The mRNA levels of *ampC* and *mexB* were normalized to those of *rpsL* for each strain and expressed as a relative ratio (fold change) compared with those of the reference strain PAO1. For each strain, three independent biological replicates in triplicate were analyzed.

### 2.10 Bacterial survival assays

*P*. *aeruginosa* strains were cultured to log phase, and cells were pelleted by centrifugation at 6,000×g for 5 min, then resuspended in sterile PBS. The initial concentration of the bacterial suspension was adjusted to approximately 10^6^ CFU/mL. For the human serum killing assay, 24 μL diluted bacterial solution was mixed with 96 μL fresh serum or sterile PBS and incubated at 37°C without shaking. 100 μL aliquots were retrieved at 2 h, followed by serial dilution and streaking onto LB agar plates for colony counting. The serum pool used in the present study was prepared from at least 5 healthy volunteers. The bacterial survival rate was expressed as the ratio of CFUs obtained after serum treatment to the CFUs treated with PBS.

For the H_2_O_2_ treatment assay, cells were treated with 50 mM H_2_O_2_ at 37°C with shaking (220 rpm) for 15 min. For the antimicrobial peptide LL-37 killing assays, cells were treated with 50 μg/mL LL-37 at 37°C with shaking (220 rpm) for 60 min. 100 μL aliquots were plated on LB agar plates to determine CFU. Bacteria treated with PBS were used as the control. These experiments were independently performed three times. The bacterial killing rate=[1−(CFU treated with H_2_O_2_ or LL-37)/(CFU treated with PBS)]×100%.

### 2.11 *Galleria mellonella* killing assays

*G. mellonella* larvae infection experiment was carried out as previously described (Yuan et al., 2019). The overnight culture of *P*. *aeruginosa* strains was inoculated into fresh LB broth at a 1:100 ratio and subsequently cultured to an OD600 of 0.5-0.6. Bacterial cells were collected by centrifugation, washed three times with sterile PBS, and serially diluted in sterile PBS. Each *G*. *mellonella* (with a body weight of 200 to 300 mg, purchased from Tianjin Huiyude Biotech Company, Tianjin, China) was injected with 10 μL of the inoculum (1×10^4^ CFU) using a 50 μL Hamilton syringe. Larvae injected with *P*. *aeruginosa* PAO1 or sterile PBS served as controls. The larvae were then incubated at 37°C, and the number of surviving larvae was counted over 3 days at 12-hour intervals.

### 2.12 Statistics analysis

Statistical analyses were performed using GraphPad Prism 10.1.2, and data were expressed as means ± standard deviation (SD) from three biological replicates. For the growth assay, pyocyanin production, bacterial survival assays, and qRT-PCR experiment, statistical significances were evaluated using one-way analysis of variance (ANOVA). For the biofilm formation and pyoverdine production assays, Brown-Forsythe and Welch ANOVA tests combined with Dunnett’s T3 multiple comparisons test were employed for statistical significance analysis. The survival curves of *G. mellonella* larvae were analyzed via a log-rank (Kaplan-Meier method) test. *p < 0.05, **p < 0.01, ***p < 0.001, and ****p < 0.0001 indicate the levels of statistical significance.

## 3 Results

### 3.1 Resistance phenotype and genomic analysis

Two *P. aeruginosa* clinical isolates, SCPa14 and SCPa16, were recovered from a single silicosis patient, with a 9-day interval between the sampling times. Antimicrobial susceptibility testing showed that both SCPa14 and SCPa16 were resistant to imipenem. SCPa14 also showed resistance to piperacillin and amikacin but intermediate to quinolones; in contrast, SCPa16 exhibited the opposite pattern, being intermediate to piperacillin and amikacin yet resistant to quinolones (**Supplementary Table S2**). The differences in antimicrobial susceptibility between the intermediate and resistant phenotypes were significant for SCPa14 and SCPa1416, with the diameters of the antimicrobial susceptibility disc inhibition zones differing by at least 5 mm, except in the case of amikacin.

WGS was performed using the HiSeq 2000 platform. According to the WGS data, SCPa14 and SCPa16 are homologous strains, and both belong to ST274, an international epidemic high-risk clone of *P. aeruginosa* (Chichón et al., 2023). ANI analysis revealed that both strains are PAO1-like, as they had 99.4% identity to the genome of reference strain PAO1, but only 98.8% identity to that of reference strain PA14. They contained identical resistance gene profiles, mediating resistance to *β*-lactams (*bla*_OXA-486_ and *bla*_PAO_), aminoglycosides (*aph(3’)-IIb*), fosfomycin (*fosA*), and chloramphenicol (*catB7*) (**Supplementary Table S2**). SCPa14 and SCPa16 also shared identical virulence gene profiles. The detection of the virulence-associated genes *exoT*, *exoY*, *exoU*, and *exoS* in the genome, which encodes cytotoxins of the type III secretion system of *P. aeruginosa* (Lee et al., 2005), revealed the presence of *exoT*, *exoY*, and *exoS*. In addition, virulence factors such as genes associated with flagella, type IV pili, secretion system, phenazines biosynthesis, alginate biosynthesis and regulation, rhamnolipid biosynthesis, phospholipase C, pyochelin, toxin, protease, and quorum sensing were also detected (**Supplementary Table S3**). Notably, we found that the *pvd* gene clusters (responsible for the synthesis of pyoverdine) and the *fpvA* gene (encoding the pyoverdine receptor) were absent from the chromosomes of strains SCPa14 and SCPa16. As pyoverdine is the main siderophore in iron-gathering capacity produced by *P. aeruginosa* (Meyer et al., 1996), the loss of pyoverdine synthesis may lead to impaired survival of bacteria under iron-limiting conditions, and, ultimately, attenuated virulence. Besides, a *pglD*-like virulence gene predicted to be involved in immune evasion was identified in SCPa14 and SCPa16, but is absent in the reference strains PAO1 and PA14. *pglD*, which encodes the UDP-4-amino acetyltransferase PglD, is required for the biosynthesis of UDP-BacAc2, the key glycosyl donor for the synthesis of bacterial surface polysaccharides (Olivier and Imperiali, 2008). The presence of the *pglD*-like gene in SCPa14 and SCPa16 may enhance bacterial virulence regulation and host-associated environmental adaptation.

**Figure 1:**
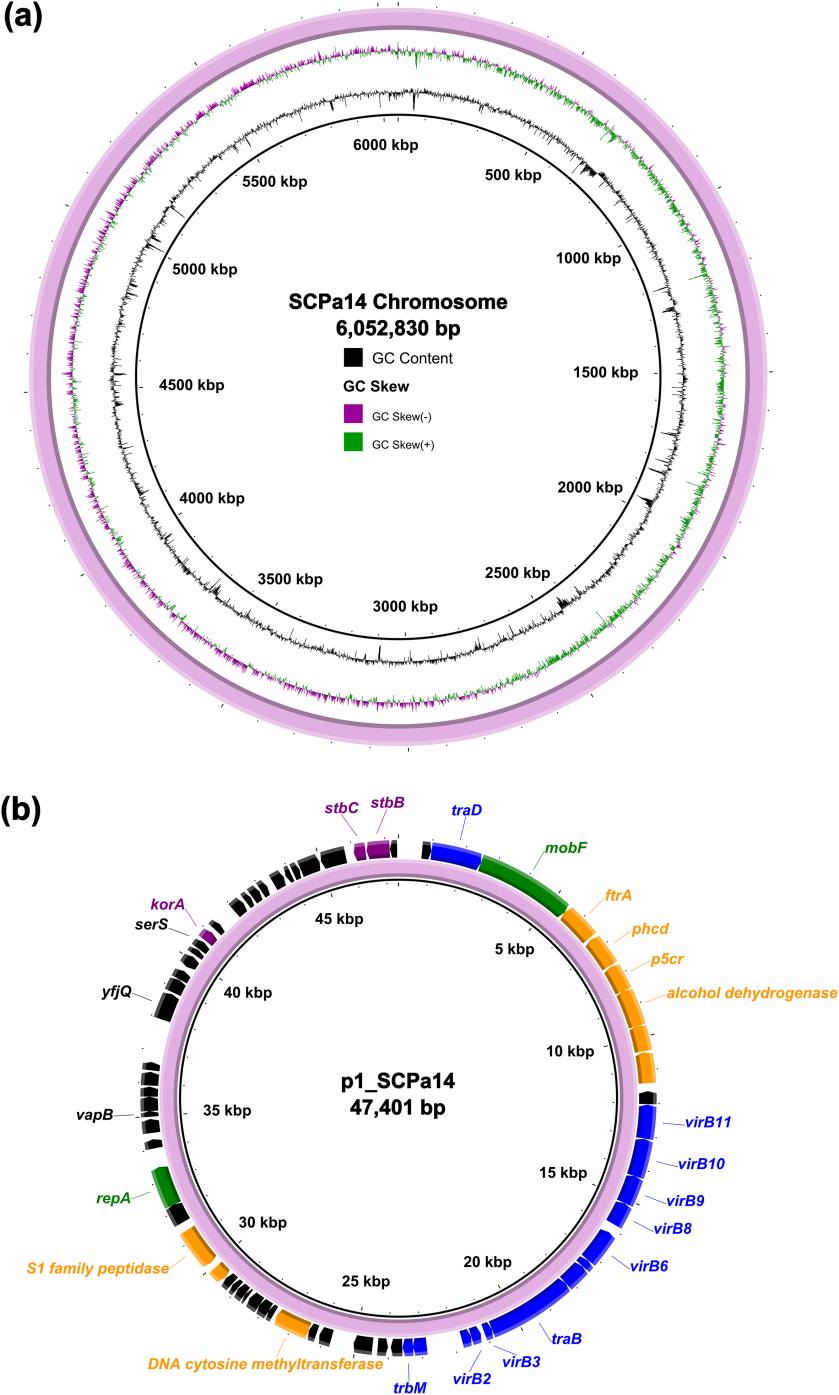
WGS analysis of *P. aeruginosa* SCPa14. **(a)** Schematic map of the chromosome of SCPa14. **(b)** Schematic map of plasmid p1_SCPa14. Genes are denoted by arrows. The backbone genes on the plasmid are highlighted in green (*repA* and *mobF*), purple (*korA* and *stbBC*), and blue (*virB*, *tra*, and *trb* genes). representative genes in the accessory region are highlighted in orange. The maps were generated using BRIG (version 0.95).

Strain SCPa14 was further subjected to WGS using the long-read MinION Sequencer, which revealed that it had a 6,052,830-bp circular chromosome with an average Guanine and Cytosine (GC) content of 66.36% (**Figure 1a**). All the resistance genes are located on the chromosome. It also contains a plasmid with a size of 47,401 bp with 60% GC content, which encodes 68 predicted open reading frames (**Figure 1b**). Sequence alignment by Blastn analysis revealed that the plasmid, designated p1_SCPa14, only showed high similarity (>90% coverage, >98% identity) to the plasmid p1_A28601 recovering from a clinical *P. aeruginosa* isolate in Germany in 2022 (Accession no. CP181555), and a region on the chromosomes of animal-derived *P. aeruginosa* strains 484919 and 488958 in United Kingdom in 2024 (Accession no. CP173128 and CP173125), suggesting that they may share the same origin. Sequence analysis revealed that the backbone of p1_SCPa14 includes regions responsible for plasmid replication (*repA* and *mobF*), maintenance (*korA* and *stbBC*), and conjugative transfer (*virB*, *tra*, and *trb* genes). In the accessory region, genes encoding energy metabolism, carbon source utilization, and environmental adaptation, such as *orf381* (encoding an alcohol dehydrogenase), *orf354* (encoding a DNA cytosine methyltransferase), and *orf408* (encoding an S1 family peptidase) were identified (**Figure 1b**).

### 3.2 Mucoidity, imipenem resistance, and genetic changes

**Figure 2:**
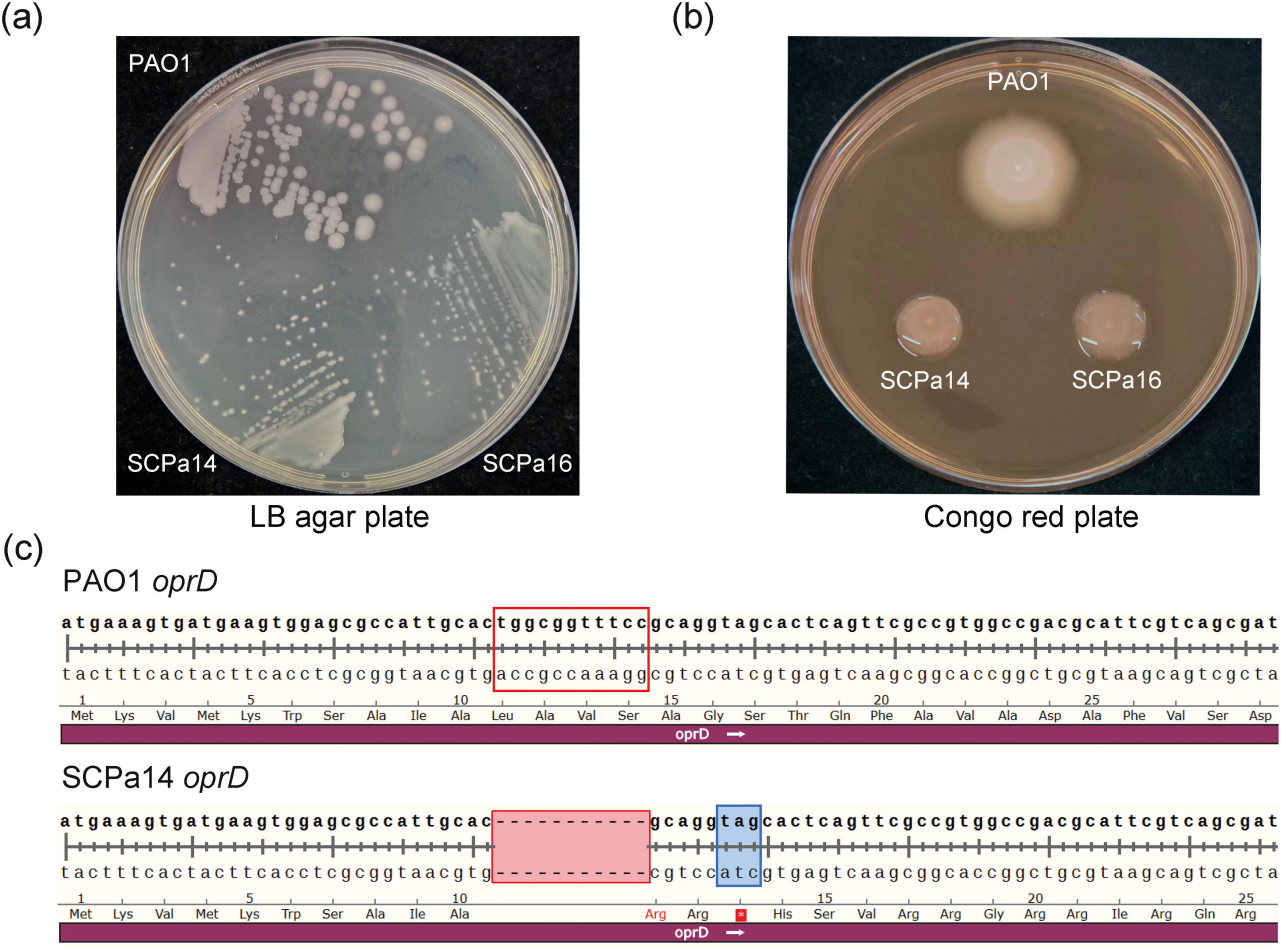
Characterization of clinical isolates SCPa14 and SCPa16. **(a)** Colony morphology of SCPa14 and SCPa16 after 36 h of growth at 37°C on LB agar plates. **(b)** Colonies morphology of SCPa14 and SCPa16 after 24 h of incubation at room temperature on Congo red plates. **(c)** Sequence alignment of the *oprD* gene between SCPa14 and PAO1. An 11-bp nucleotide deletion (positions 32–42 nt) in the *oprD* gene of strain SCPa14 resulted in the formation of a premature stop codon. The deleted 11-bp nucleotides were marked with a red border, while the premature stop mutation was highlighted with a blue shadow.

Sub-cultivation of SCPa14 and SCPa16 on LB agar plates yielded a small-colony-variant phenotype relative to the *P. aeruginosa* reference strain PAO1 (**Figure 2a**). On Congo red plates, both isolates formed mucoid colonies, indicating the overproduction of the exopolysaccharide alginate (**Figure 2b**). The emergence of mucoid alginate-overexpressing morphotypes in *P. aeruginosa* is typically associated with mutations in the *mucA* gene (Moyano et al., 2007). As both SCPa14 and SCPa16 exhibited a mucoid morphotype, we analyzed their *mucA* gene sequences. Compared to the *mucA* sequence of PAO1, two mutations were identified in both SCPa14 and SCPa16: a synonymous mutation (CAA→CAG) at nucleotide position 342, and a G deletion at nucleotide position 433 (ΔG433). Previous studies have demonstrated that loss-of-function mutations in *mucA*, such as the well-characterized *mucA22* mutation, are causative for mucoidy in clinical CF isolates of *P. aeruginosa* (Bragonzi et al., 2006). Here, the mucoid alginate-overexpressing morphotype observed in SCPa14 and SCPa16 is probably driven by the frameshift mutation ΔG433 in *mucA.*

Several carbapenem resistance mechanisms have been identified in *P. aeruginosa*, including reduced carbapenem influx caused by porin protein OprD deficiency, carbapenemase release, increased activity of *ampC*, and efflux pump overexpression (Quale et al., 2006; Li et al., 2012). To explain the imipenem resistance in SCPa14 and SCPa16, we determined the expression levels of the *ampC* β-lactamase gene and the *mexB* multidrug efflux gene by qRT-PCR. Unexpectedly, both strains exhibited no overexpression of *mexB* or *ampC* compared with the corresponding levels in the reference strain PAO1 (**Supplementary Figure S1**). OprD, encoded by 443 amino acids, is a porin important for the entry of carbapenems and is considered a primary mechanism underlying carbapenem resistance in *P. aeruginosa* (Li et al., 2012). We detected alterations in the *oprD* gene in SCPa14 and SCPa16, which showed a frameshift mutation due to an 11-bp nucleotide deletion (positions 32 to 42 nt) in *oprD*, resulting in a premature stop codon at amino acid position 13 (**Figure 2c**). The loss of OprD may be responsible for the imipenem resistance in SCPa14 and SCPa16.

Genomic comparative analysis showed that SCPa14 and SCPa16 only differed by 11 SNPs and an insertion mutation between their whole genomes (**Supplementary Table S4**). Among these SNPs, 9 were predicted to impact the function of the encoded proteins, and the corresponding genes mainly include those related to iron metabolism (*hemR*, *fdx*, *tdhA*, and *tolQ*), fatty acid synthesis (*fabD* and *fabG*), porin-like protein NicP (*nicP*), GTP pyrophosphokinase (*relA*), and DNA topoisomerase IV (*parE*). Especially, the missense mutation (D419N, GAC→AAC) in the *parE* gene may reduce the binding affinity of DNA topoisomerase IV (the protein encoded by *parE*) to quinolones, thereby decreasing the susceptibility of strain SCPa16 to these antibiotics (Pang et al., 2019). By contrast, a premature stop codon (Q283*, CAG→TAG) is present in the *relA* gene of strain SCPa14, but not in SCPa16, which shortens the RelA protein from its wild-type length of 747 amino acids (aa) to only 282 aa. In *P. aeruginosa* PAO1, RelA functions as a GTP pyrophosphokinase that acts as both a key (p)ppGpp synthetase and a stringent response regulator (Erickson et al., 2004). The Q283* mutation in *relA* is thus hypothesized to reduce (p)ppGpp synthesis in SCPa14, which could consequently increase the bacterial susceptibility to quinolones (Pacios et al., 2020). Taken together, sequence variations in the *parE* and *relA* genes can jointly account for the higher quinolone resistance exhibited by SCPa16 compared with SCPa14. Previous studies also showed that RelA contributes to bacterial resistance to multiple environmental stresses and is required for full virulence of *P. aeruginosa* (Yang et al., 2019). Truncation of the *relA* gene in SCPa14 could affect bacterial adaptation and pathogenicity in the host.

### 3.3 Host adaptation and virulence

**Figure 3:**
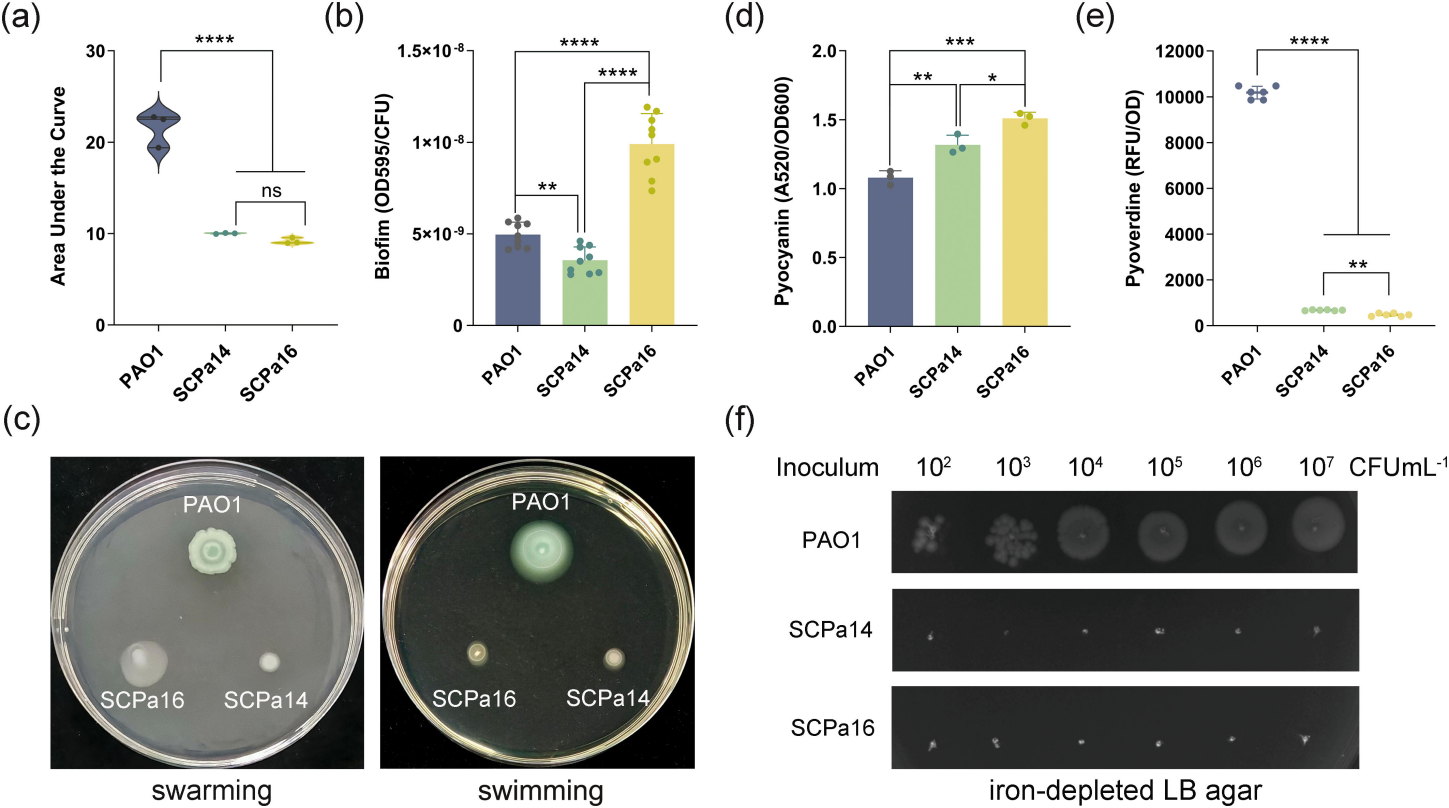
Virulence-associated characteristics of SCPa14 and SCPa16. **(a)** Growth assay. The growth curves were converted into AUC (Area Under the Curve) by GraphPad Prism 10.1.2 to evaluate the growth abilities. The growth curves of *P*. *aeruginosa* strains are presented in **Supplementary Figure S2**. **(b)** Crystal violet staining for the quantification of biofilm formation. **(c)** Representative images of swarming and swimming. **(d)** Quantitation of pyocyanin. **(e)** Quantitation of pyoverdine. Data are presented as the mean ± SD from at least three independent experiments in triplicate. Statistical analyses were evaluated using one-way ANOVA **(a, d)** or Brown-Forsythe and Welch ANOVA tests followed by Dunnett’s T3 multiple comparisons test **(b, e)**. *p < 0.05, **p < 0.01, ***p < 0.001, and ****p < 0.0001. **(f)** Growth of *P*. *aeruginosa* strains on the iron-limited LB agar plates.

Chronic respiratory tract infection with *P. aeruginosa* typically exhibits extensive phenotypic adaptation to the respiratory tract environment. Growth kinetic assays showed that both SCPa14 and SCPa16 grew significantly more slowly in LB medium compared with the reference strain PAO1 (*p* < 0.0001, **Figure 3a**). However, SCPa16 formed significantly more abundant biofilm than that of SCPa14 and PAO1 after 48h-inoculation (*p* < 0.0001, **Figure 3b**). The motility assay revealed reduced swimming and swarming in both SCPa14 and SCPa16 compared to PAO1, while SCPa16 exhibited higher swarming than SCPa14 (**Figure 3c**). Pyocyanin, an electrochemically active metabolite of *P. aeruginosa*, is recognized as a virulence factor in chronic lung infections in CF patients (Das and Manefield, 2012). In this study, clinical isolates SCPa14 and SCPa16 exhibited a significant enhancement in pyocyanin synthesis than that of PAO1 (*p* = 0.0046 and *p* = 0.0002, respectively, **Figure 3d**). Moreover, SCPa16 produced more pyocyanin than SCPa14 (*p* = 0.0131).

Siderophores production is one of the strategies used by *P. aeruginosa* to uptake iron, which is a key element for bacterial adaptation and pathogenesis in the host (Ghssein and Ezzeddine, 2022). Pyoverdines are the primary siderophores that are synthesized under low iron conditions. Here, we found that SCPa14 and SCPa16 produced significantly less pyoverdine than that of PAO1 (both, *p* < 0.0001, **Figure 3e**). This is consistent with the genetic analysis, which reveals that pyoverdine-related genes are lost in SCPa14 and SCPa16. Corresponding to this, the growth of SCPa14 and SCPa16 was completely inhibited on the iron-depleted LB agar (supplemented with 100 μM 2, 2-dipyridyl) (**Figure 3f**), indicating a significant reduction in their iron chelation ability.

**Figure 4:**
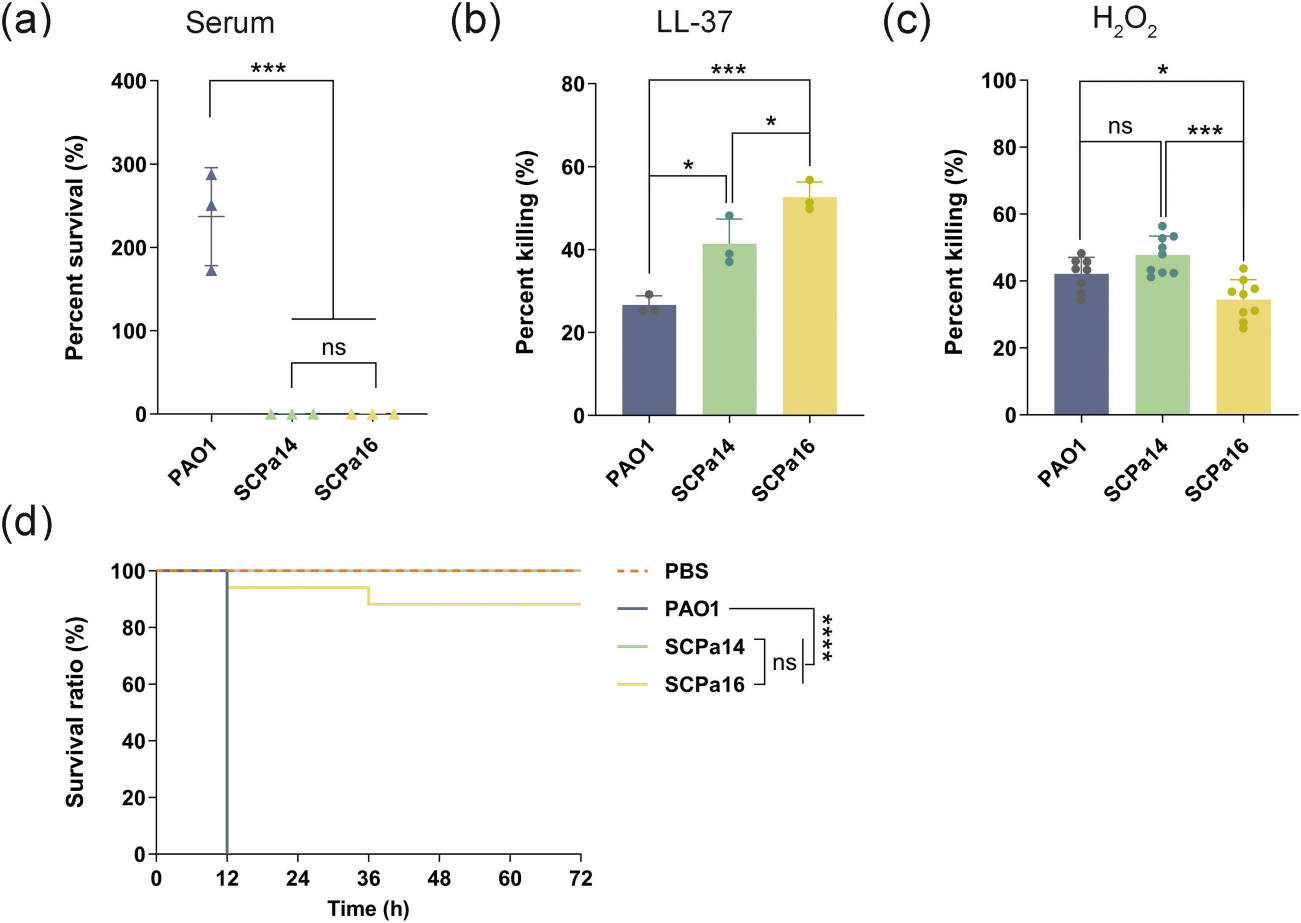
The adaption and virulence of SCPa14 and SCPa16. Log-phase cultures of *P*. *aeruginosa* strains were treated with human serum **(a)**, LL-37 **(b)**, and H_2_O_2_ **(c)**. Bacteria treated with PBS were used as the control. CFUs were quantified after treatment and survival or killing rate was determined. Data represent the mean ± SD from three independent experiments in triplicate. Statistical analyses were performed using one-way ANOVA. **(d)** The survival of *G*. *mellonella* larvae. Each larva was injected with 10 μL of *P. aeruginosa* dilution (1×10^4^ CFU) or PBS (negative control). The larvae were monitored for 72 h after the infection. A log-rank (Kaplan-Meier method) test was employed for statistics, *p < 0.05, ***p < 0.001, ****p < 0.0001, and ns, not significant.

To determine whether there is an advantage of SCPa14 and SCPa16 in evading host effectors, we exposed them to either human serum, antimicrobial peptide LL-37, or H_2_O_2_. Results showed that SCPa14 and SCPa16 lack resistance to serum-mediated killing; after 2 hours of culture in medium containing 80% human serum, their survival rate were 7.90×10^-5^ ± 6.99×10^-5^% and 1.12×10^-3^ ± 1.29×10^-3^%, respectively, significantly lower than that of PAO1 (237.00 ± 58.82%, both, *p* = 0.0003, **Figure 4a**). Similarly, SCPa14 and SCPa16 were susceptible to LL-37; after treatment with 50 μg/mL LL-37 for 1h, 41.41 ± 5.94% of SCPa14 and 52.65 ± 3.66% of SCPa16 were killed, with significantly higher mortality than PAO1 (26.62 ± 2.24%, SCPa14 vs PAO1, *p* = 0.0123; SCPa16 vs PAO1, *p* = 0.0007, **Figure 4b**). Even so, SCPa14 exhibited better resistance to LL-37 than SCPa16 (*p* = 0.0399). In contrast, SCPa16 exhibited more resistance to H_2_O_2_; upon exposure to 50 mM H_2_O_2_ for 15 min, the killing percentage of SCPa16 was 34.45 ± 5.97%, significantly lower than that of SCPa14 (47.77 ± 5.65%, *p* = 0.0001) and PAO1 (42.12 ± 4.92%, *p* = 0.0241, **Figure 4c**).

A *G. mellonella* larvae infection model was employed to evaluate the virulence of SCPa14 and SCPa16. In this model, larvae infected with 1*10^4^ CFU of the reference strain PAO1 exhibited high mortality rates, reaching 100% at 12 h post-infection. In contrast, SCPa14 and SCPa16 significantly decreased larval mortality compared to PAO1 (*p* < 0.0001, **Figure 4d**). By 72 h post-infection, SCPa16 only led to 20% larval mortality, whereas SCPa14 caused no detectable larval death throughout the 72-h observation period. These results indicate that SCPa14 and SCPa16 are low-virulence strains.

## 4 Discussion

*P. aeruginosa* is a notorious cause of acute and chronic infections in hospital settings. In this study, two homologous ST274-*P. aeruginosa* strains, SCPa14 and SCPa16, were isolated from the sputum sample of a single silicosis patient with an interval of 9 days. *P. aeruginosa* ST274 is known as an epidemic multidrug-resistant clone associated with chronic infections and intrabody adaptive evolution in CF patients in Europe (Fernández-Olmos et al., 2013). It is also one of the two most common STs among carbapenem-resistant *P. aeruginosa* isolates in Japan (Yano et al., 2024). In the present study, imipenem resistance was identified in SCPa14 and SCPa16, and this resistance was probably attributed to the combined effects of slow growth and a frameshift mutation in *oprD* in our case. We also observed elevated quinolone resistance in SCPa16 compared with SCPa14, and the genetic variations in *parE* (the ciprofloxacin target-encoding gene) and *relA* (the (p)ppGpp synthetase gene) may be correlated with this phenotypic change. Interestingly, we noticed a trade-off in antibiotic resistance evolution in SCPa16, where the acquisition of quinolone resistance leads to an increased susceptibility to piperacillin and amikacin. Previous studies have also described the collateral sensitivity (CS) between ciprofloxacin and aminoglycosides, aztreonam, or colistin in *P. aeruginosa* (Imamovic et al., 2018; Hernando-Amado et al., 2023). Given that ciprofloxacin is frequently used to treat *P. aeruginosa* infections (Rehman et al., 2019), the identification of robust ciprofloxacin-based CS patterns thus holds important implications for optimizing practical antibiotic regimens.

It has been proposed that reduced growth rates and mucoid conversion are hallmarks of well-adapted *P. aeruginosa* strains within infected hosts (Li et al., 2005; Yang et al., 2008), and loss of motility could be beneficial over the course of infection (Amiel et al., 2010). Compared to *P. aeruginosa* PAO1, both SCPa14 and SCPa16 exhibited a chronic infection phenotype characterized by mucoid, slow growth, and reduced motility, which could confer selective advantages in the context of persistent lung infections. Biofilms are responsible for many persistent and chronic infections and are particularly important for *P. aeruginosa* pathogenesis (Thi et al., 2020). Not surprisingly, the mucoid SCPa16 exhibited significantly higher biofilm-forming ability compared to PAO1, as alginates are one of the exopolysaccharides produced by *P. aeruginosa* that contribute to biofilm architecture. However, SCPa14, which also exhibited a mucoid morphotype, showed impaired ability to produce biofilm. This may be due to a premature termination mutation in the (p)ppGpp synthetase gene *relA* in SCPa14, as the stringent response was demonstrated to positively regulate biofilm-associated pathways in *P. aeruginosa* (Schafhauser et al., 2014; Engelhardt et al., 2025). In addition, the mutated RelA in SCPa14 could also contribute to its impaired swarming motility, as supported by a previous study that demonstrated a close link between (p)ppGpp and cell viability (Xu et al., 2016; Engelhardt et al., 2025).

Pyocyanin is an important virulence factor of *P. aeruginosa* and one of the key mechanisms underlying the bacterium’s ability to establish and persist in chronic infections (Lau et al., 2004). It has been established that the inflammatory lung environment can induce the secretion of pyocyanin by *P. aeruginosa* (Caldwell et al., 2009). The enhanced pyocyanin synthesis in SCPa14 and SCPa16 was compatible with the evolutionary pressure for its adaptation to the host’s stressful environment. In addition to its well-documented role as an iron-scavenger, the siderophore pyoverdine is also implicated in the virulence regulation and pathogenic potential of *P. aeruginosa* (Lamont et al., 2002). Those pyoverdine-deficient *P. aeruginosa* mutants have a greatly reduced ability to cause disease in animal models (Minandri et al., 2016). Conversely, it has also been demonstrated that *P. aeruginosa* mutants that are unable to synthesize pyoverdine exhibit enhanced adaptation to the CF airway environment, as pyoverdine-negative mutants can exploit individuals that produce the energetically costly iron-scavenging siderophore (Winstanley et al., 2016). For instance, in a study analyzing sputum samples from CF patients, pyoverdine was not detected in 21 of the 114 samples in which *P. aeruginosa* was present (Martin et al., 2011). In this study, all pyoverdine synthesis genes (*pvd* gene cluster) and their receptor gene *fpvA* were absent in these two *P. aeruginosa* isolates from a silicosis patient, with the corresponding phenotype characterized by extremely low pyoverdine levels. This lack of pyoverdine would attenuate strain virulence potential while concomitantly increasing their risk for persistent host infection.

We propose that the mucoid morphotype and enhanced pyocyanin production of SCPA14 collectively facilitate its initial airway colonization. Meanwhile, the lack of (p)ppGpp caused by the *relA* mutation may significantly reduce the adaptability and virulence potential of this strain. For SCPa16, in comparison to SCPa14, it retains a complete capacity to synthesize (p)ppGpp, thereby forming a more robust biofilm, exhibiting elevated quinolone resistance, producing higher pyocyanin levels, and showing higher cell motility, all of which facilitate its better survival and persistence in the host environment (Xu et al., 2016). However, despite harboring a large number of virulence genes, both strains exhibited slow growth and poor resistance to some host-derived stress environments (such as, iron-restriction) as well as innate antimicrobials (such as serum complement and LL-37), rendering them low-virulence strains, as evident from the results of the *G. mellonella* infection experiment.

We are aware of the limitations of the bacterial isolation method employed in this study, due to which we were unable to distinguish between the parental and evolved strains of these two isolates, nor could we exclude the possibility of their coexistence in the respiratory tract. However, a premature stop codon (Q283*) is present in the *relA* gene of strain SCPa14, but is absent in that of SCPa16, which indicates, at the very least, that strain SCPa16 is not directly evolved from SCPa14. The existing data also do not provide sufficient evidence to support the hypothesis that SCPa14 is an evolutionary derivative of SCPa16. Given that phenotypic assays demonstrated that SCPa14 has a weaker potential for environmental adaptation than SCPa16, we hypothesize that both SCPa14 and SCPa16 may have evolved from a common ancestral strain in this patient, coexisting within the host environment. Subsequently, SCPa14 was likely outcompeted due to its inferior adaptive capacity, thereby enabling SCPa16 to gradually emerge as the dominant strain.

In conclusion, our study demonstrated genotypic and phenotypic characteristics of two homologous *P. aeruginosa* isolates and highlighted their adaptive strategies in the stressful conditions during airway infection in the silicosis patient. The findings may provide new clues for the development of interference strategies against *P. aeruginosa* infections in silicosis patients.

## Data Availability

The whole genome sequences of SCPa14 and SCPa16 have been deposited into the public GenBank database under the project no. PRJNA1355304.
