# Supplementary material for: Within-host adaptation mutations associated with persistent colonization of Pseudomonas aeruginosa in a silicosis patient
Source: Front Cell Infect Microbiol. 2026 Feb 17;16:1739179. doi: 10.3389/fcimb.2026.1739179 (PMC12953531; doi:10.3389/fcimb.2026.1739179)
Supplement: Supplementary file 2 [file Table2.docx]

Supplementary Material

**Table S1 Primer sequences for qRT-PCR.**

| **Primer** | **Sequence (5’-3’)** | **Product length (bp)^a^** | **Reference** |
| --- | --- | --- | --- |
| *mexB*-F | CAAGGGCGTCGGTGACTTCCAG | 273 | [1] |
| *mexB*-R | ACCTGGGAACCGTCGGGATTGA |  |  |
| *ampC*-F | GCTGGGAAGCCTACGACTGG | 240 | This study |
| *ampC*-R | CCCGCTCGGCATTGGGATAG |  |  |
| *rpsL*-F | GCAAGCGCATGGTCGACAAGA | 201 | [2] |
| *rpsL*-R | CGCTGTGCTCTTGCAGGTTGTGA |  |  |

^a^bp, base pair.

**Table S2 Resistance gene profiles and phenotypes of *P. aeruginosa* SCPa14 and SCPa16.**

| **Strain** | **Antimicrobial resistance genes** | **Resistance phenotype^a, b^** | | | | | | | | | | | | | |
| --- | --- | --- | --- | --- | --- | --- | --- | --- | --- | --- | --- | --- | --- | --- | --- |
|  |  | **PIP** | **PTZ** | **CS** | **CAZ** | **FEP** | **ATM** | **IPM** | **MEM** | **GEN** | **TOB** | **AMK** | **LVX** | **CIP** |  |
| SCPa14 | *bla*_OXA-486_, *bla*_PAO_, *aph*(*3'*)*-IIb*, *fosA*, *catB7* | **R(11)** | S(23) | S(37) | S(18) | S(20) | S(25) | R(11) | S(21) | S(17) | S(18) | **R(14)** | I(15) | I(19) |  |
| SCPa16 | *bla*_OXA-486_, *bla*_PAO_, *aph*(*3'*)*-IIb*, *fosA*, *catB7* | I(18) | S(23) | S(26) | S(25) | S(21) | S(29) | R(12) | S(19) | S(16) | S(15) | I(15) | **R(8)** | **R(13)** |  |

^a^ATM, aztreonam; AMK, amikacin; CAZ, ceftazidime; CIP, ciprofloxacin; CS, cefoperazone/sulbactam; FEP, cefepime; GEN, gentamicin; IPM, imipenem; LVX, levofloxacin; MEM, meropenem; PIP, piperacillin; PTZ, piperacillin/tazobactam; TOB, tobramycin.

^b^R, resistant; S, susceptible; I, intermediate. Values in parentheses indicate inhibition zone diameters (Kirby-Bauer method).

**Table S4 Summary of genetic mutations in SCPa16 relative to SCPa14.**

| **Gene name** | **Mutation** | **Putative gene function** |
| --- | --- | --- |
| *fabD* | A→C, S75R (AGC→CGC) | Malonyl CoA‑acyl carrier protein transacylase |
| *fabG* | A→C, D169A (GAC→GCC) | 3‑oxoacyl‑[acyl‑carrier‑protein] reductase FabG |
| *hemR* | T→G, N344T (AAC→ACC) | Hemin receptor |
| *hemR/hemS* | T→C, intergenic (‑128/‑53) | Hemin receptor/Hemin transport protein HemS |
| *fdx* | G→A, V90M (GTG→ATG) | 2Fe‑2S ferredoxin |
| *relA* | T→C, *283Q (TAG→CAG) | GTP pyrophosphokinase |
| *tolQ* | G→C, E205Q (GAG→CAG) | Tol‑Pal system protein TolQ |
| *tdhA* | T→C, I369T (ATC→ACC) | TonB‑dependent heme receptor A |
| *nicP* | G→T, L17L (CTG→CTT) | Porin‑like protein NicP |
| *parE* | G→A, D419N (GAC→AAC) | DNA topoisomerase 4 subunit B |
| *cntO/mntP* | G→T, intergenic (‑304/‑333) | Metal‑pseudopaline receptor CntO/putative manganese efflux pump MntP |
| *AHKBIJOD_04351* | +GGTCAGCGCGC  coding (1163/1248 nt) | hypothetical protein |


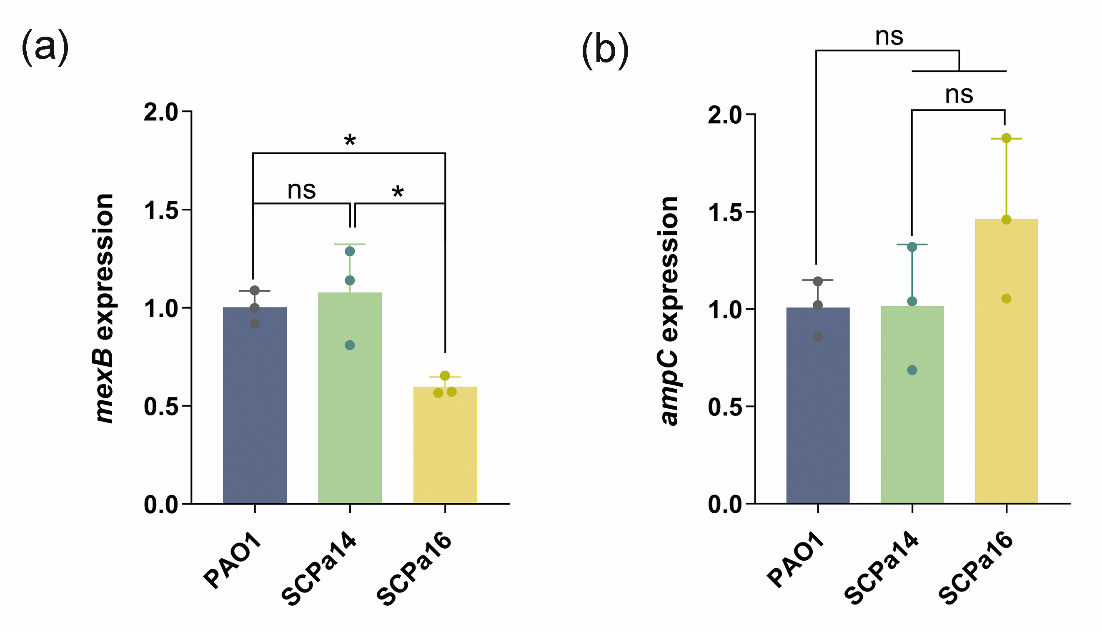


**Fig. S1** qRT-PCR analysis for gene expression of *ampC* and *mexB*. Data represent the mean ± SD from three independent experiments performed in triplicate. Statistical analyses were performed using one-way ANOVA. **p* < 0.05, and ns, not significant.


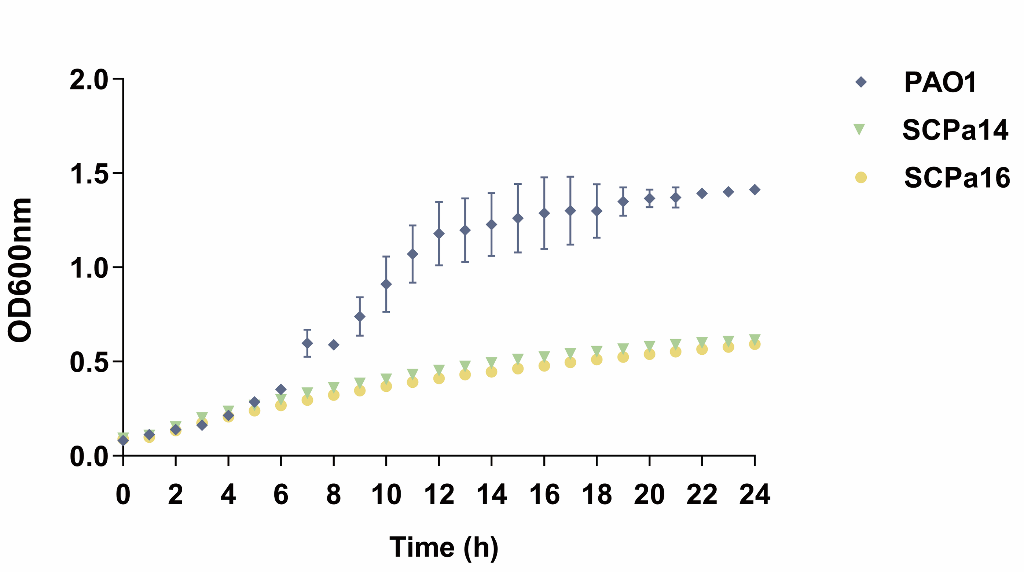


**Fig. S2** Growth curves of *P*. *aeruginosa* strains in LB broth. The culture was grown at 37°C for 24 h in 96-well plates, with a shaking cycle every 60 min when OD600 was measured.

**References**

[1] Yin L, Bao Z, He L, Lu L, Lu G, Zhai X, Wang C. Virulence factors, molecular characteristics, and resistance mechanisms of carbapenem-resistant *Pseudomonas aeruginosa* isolated from pediatric patients in Shanghai, China. BMC Microbiol. 2025 Mar 11;25(1):130. doi: 10.1186/s12866-025-03856-1.

[2]Dumas JL, van Delden C, Perron K, Köhler T. Analysis of antibiotic resistance gene expression in *Pseudomonas aeruginosa* by quantitative real-time-PCR. FEMS Microbiol Lett. 2006 Jan;254(2):217-25. doi: 10.1111/j.1574-6968.
